# Gender-specific factors affecting changes in physical function among older adults during the COVID-19 pandemic

**DOI:** 10.1038/s41598-025-16990-7

**Published:** 2025-09-01

**Authors:** Kaori Teraoka, Taishi Tsuji, Takafumi Monma, Namhoon Lim, Tomohiro Okura

**Affiliations:** 1 Department of Physical Therapy, School of Health Sciences, Japan University of Health Sciences, 2-555, Hirasuga, Satte-City, Saitama 340-0145 Japan; 2 Institute of Health and Sport Sciences, University of Tsukuba, 1-1-1 Tennodai, Tsukuba-City, 305-8577 Japan; 3 International Institute for Integrative Sleep Medicine, University of Tsukuba, Tsukuba-City, 305-8575 Japan

**Keywords:** COVID-19, Older adults, Physical function, Gender differences, Longitudinal study, Health care, Risk factors

## Abstract

This longitudinal study investigated gender-specific factors associated with changes in physical function among community-dwelling older adults during the COVID-19 pandemic. Although the impact of behavioral restrictions on older adults has been previously studied, few studies have examined individual-level longitudinal changes, especially with a focus on gender differences. A total of 242 older adults in Japan (111 men and 131 women) were followed from 2019 to 2021. Physical function was assessed using the Timed Up and Go (TUG) test and 5-m habitual walking speed. Associations between individual characteristics—such as education level (years), economic status, daily activity levels, and living arrangement—and changes in physical function were examined using linear mixed-effects models, adjusting for age, education level (years), economic status, and living arrangement. The results showed that among men, having less than 12 years of education level (years) and a higher pre-pandemic leisure activity score were significantly associated with a decline in TUG performance. Among women, living alone was associated with improved 5-m walking speed. These findings indicate that physical function changes during the pandemic varied by gender and were influenced by individual-level factors. The results highlight the importance of developing gender-sensitive and context-specific strategies to support older adults in maintaining physical function during public health emergencies.

## Introduction

The COVID-19 pandemic has imposed behavioral restrictions across all age groups, significantly altering daily life. Older adults, in particular, were disproportionately affected due to their heightened risk of severe infection and voluntary refraining from going out. Compared with younger populations, they experienced a marked reduction in physical activity (PA)^1^. Such behavioral changes have been shown to negatively impact the health and lifestyle of older adults, contributing to reduced physical activity, lower limb functional decline, and progression of frailty^2–4^. Indeed, functional deterioration during the pandemic has been associated with impaired walking ability, slower performance on the Timed Up and Go (TUG) test, reduced balance, and decreased flexibility of both upper and lower extremities^3,5^.

Several studies have also examined individual characteristics that may increase vulnerability to the effects of staying at home. For example, individuals with a BMI ≤ 22, limited social interaction, or those living alone were more likely to exhibit adverse nutritional behaviors such as decreased food intake and weight loss^6^. In frail older adults, substantial declines in leg strength and daily activity level have also been reported^7^. However, many of these prior studies relied on subjective measures such as questionnaires to assess physical function, raising concerns regarding the reliability and validity of their findings^8^. Furthermore, most have focused on population-level trends rather than tracking longitudinal changes at the individual level.

In addition, little attention has been paid to potential gender differences in the impact of the pandemic on physical function. Given biological, behavioral, and social distinctions between men and women, their vulnerability and responses to lifestyle changes may differ^9,10^. For instance, women tend to live longer than men, are more likely to live alone, and differ in how they engage in social and physical activities. These differences may influence changes in physical function under behavioral restrictions. Thus, investigating gender differences is essential for developing tailored intervention strategies.

Against this background, the present study conducted a three-year longitudinal assessment (2019–2021) of physical function in community-dwelling older adults in Japan, spanning the pre-pandemic and pandemic periods, to investigate the older adult population more likely to experience differential impacts based on individual characteristics, especially gender. Using repeated, objective measurements of physical function, we aimed to clarify how the pandemic influenced physical function and to identify gender-specific individual factors associated with these changes.

## Methods

### Data source and study participants

This study is part of the prospective cohort study known as the Kasama Study, conducted in Kasama City, Ibaraki Prefecture, Japan. The Kasama Study is a medium-scale, long-term longitudinal cohort launched in 2008 that focuses on health, physical function, and physical activity (PA) among older adults. Kasama City is a relatively small rural municipality located in central Ibaraki. Details of the Kasama Study have been reported elsewhere^9^.

**Fig. 1:**
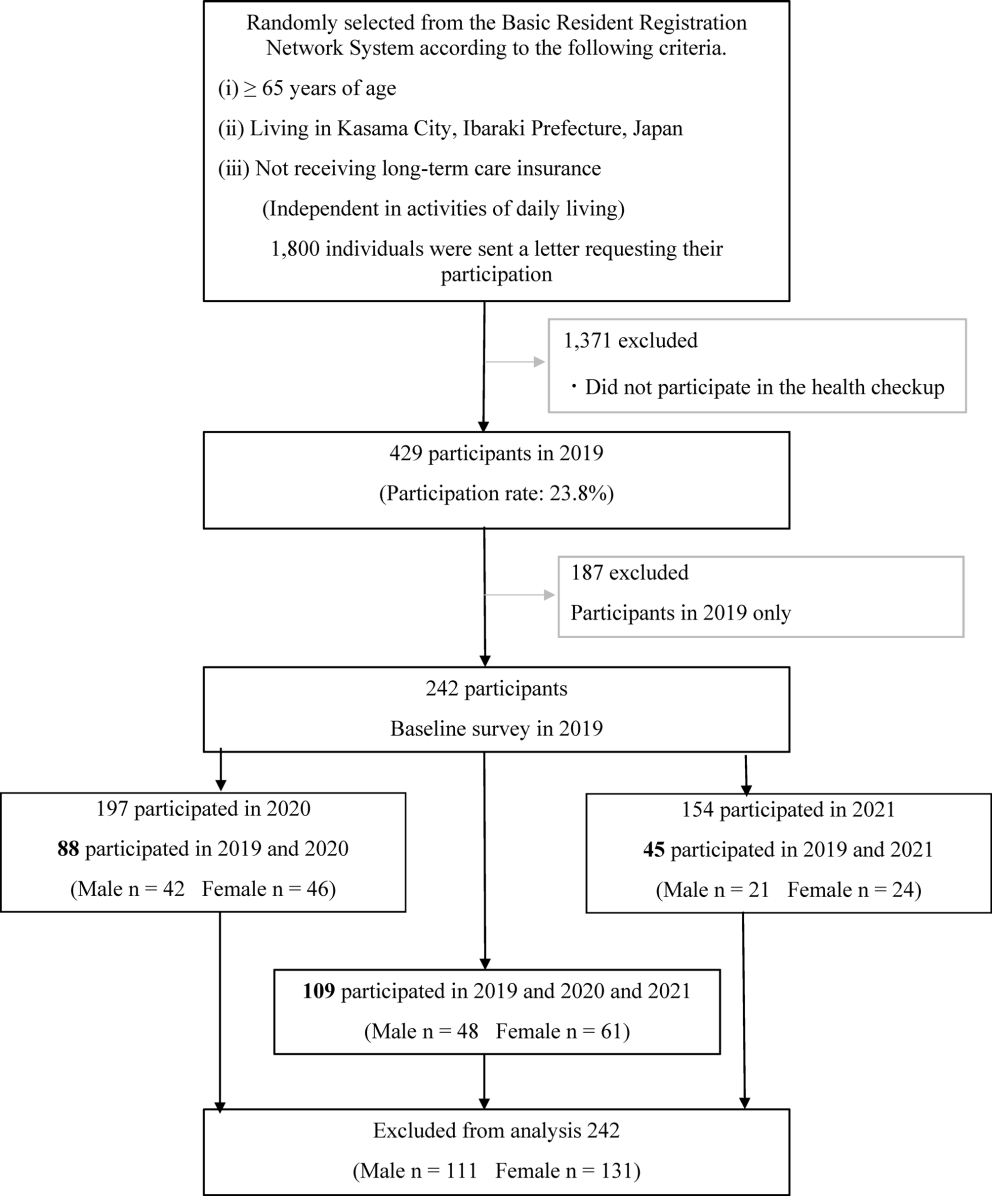
Flowchart of participants.

Figure 1 presents the participant flowchart. Individuals were randomly selected from the Basic Resident Registration Network System according to the following criteria: (i) aged 65 years or older, (ii) residing in Kasama City, and (iii) not receiving long-term care insurance services (i.e., independent in activities of daily living). In 2019, 1,800 invitation letters were distributed, with 429 individuals (23.0%) participating. In 2020, 840 individuals were contacted, and 252 (30%) participated. In 2021, 1,642 were contacted, and 257 (16%) participated. The analytical sample comprised 242 individuals, including 88 who participated in both 2019 and 2020, 45 who participated in both 2019 and 2021, and 109 who participated in all three waves (2019, 2020, and 2021). Participants who took part only in the 2019 survey (n = 187) were excluded from the analysis. The final sample consisted of 111 men and 131 women, with a mean age of 75.0 ± 5.1 years, of whom 54.1% were female.

### Survey items

#### Outcome variables: physical function tests

Five physical function tests were selected from the New Fitness Test conducted by the Ministry of Education, Culture, Sports, Science and Technology (MEXT), Japan^10^. These included handgrip strength (upper limb strength), seated forward bend (flexibility), Timed Up and Go (TUG) test (functional mobility), 5-m habitual walking speed (gait ability), and Hand working with pegboard (manual dexterity). Detailed measurement procedures are described by Tsunoda et al.^11^.

#### Explanatory variables: individual factors

Based on previous studies^11–15^, nine individual factors related to health and physical function in older adults were assessed: age, education level (years), living alone, cognitive function, social isolation, PA (total score on the Physical Activity Scale for the Elderly [PASE]), leisure activity score, household activity score, and work-related activity score. These factors were assessed using self-administered questionnaires, with 2019 as the baseline.

Participants were categorized into binary groups based on predefined cutoff values for each individual factor: 1 = at risk, 0 = not at risk. The classifications were as follows:

*Age*: 75 years or older = 1; 65–74 years = 0.

*Education level (years)*: Less than 12 years = 1; 12 years or more = 0.

*Economic status*: Based on a 5-point self-rating scale (1 = very poor to 5 = very comfortable); scores of 1–2 were coded as 1 (economically disadvantaged), and 3–5 as 0 (economically comfortable). Among women, only eight were classified as disadvantaged; thus, economic status was treated as a covariate in the analysis for women.

*Living arrangement*: Living alone = 1; living with others = 0. Among men, only four were living alone; thus, living arrangement was treated as a covariate in the analysis for men.

*PA scores*: PASE^16^ total, leisure, household, and work-related activity scores were used. Low activity levels were coded as 1, high activity as 0.

*Cognitive function*: Assessed using the Five-Cognitive Function Test (Five-Cog)^17^; low scores indicating cognitive decline were coded as 1, high scores as 0.

*Social isolation*: Assessed using the Japanese version of the Lubben Social Network Scale–Short Form (LSNS-6)^18^; scores below 12 (indicative of isolation) were coded as 1, and 12 or above as 0.

Appropriate permissions and licenses were obtained for the use of all copyrighted scales in this study. Specifically, licenses for the Physical Activity Scale for the Elderly (PASE) and the Five-Cognitive Functions Test (Five-Cog) were purchased prior to data collection. The Geriatric Depression Scale (GDS) and the Lubben Social Network Scale (LSNS-6) are publicly available for academic research purposes, and their use complied with the developers’ terms of use.

### Statistical analysis

To account for the non-independence of repeated measurements collected from the same participants over multiple years, a linear mixed-effects model was used. A random intercept was included for each participant, and year (2019, 2020, 2021) was treated as a fixed effect to appropriately model within-subject correlations over time. Values presented in Tables 2, 3, and 4 represent data aggregated by year, including all participants for each year, even those who participated in multiple years.

For the nine individual factors, binary variables (1 = at risk, 0 = not at risk) were created based on predefined cutoff values. To examine whether changes in physical function tests over time differed by risk status, a linear mixed-effects model including an interaction term between group (risk vs. non-risk) and year was employed. All models included the year 2019 and the low-risk group as reference categories.

The model accounted for the nested structure of repeated physical function measures (level 1) within individuals (level 2). All analyses were adjusted for age, education level (years), economic status, and living arrangement. For each covariate under examination, the remaining three variables were included as adjustment factors.

All analyses were conducted using Stata/SE (StataCorp, College Station, Texas, USA). To correct for multiple comparisons, Bonferroni adjustment was applied, and statistical significance was set at p < 0.0056 (0.05 ÷ 9 factors).

### Ethical considerations

This study was conducted in accordance with the principles of the Declaration of Helsinki and was approved by the Ethics Committee of the Faculty of Health and Sport Sciences, University of Tsukuba (Approval No. 30-5). The purpose, methods, and handling of personal data were explained to participants both in writing and verbally, and written informed consent was obtained from all participants.

## Results

Table 1 presents the baseline characteristics of the participants by gender at the time of the 2019 survey. Nine individual factors (age, education level (years), living arrangement, cognitive function, social isolation, and the PASE total score, leisure activity score, household activity score, and work-related activity score) were classified into two categories based on predefined cutoff values associated with risk of physical function decline (e.g., age ≥ 75 vs. 65–74; education level (years) < 12 vs. ≥ 12 years). These classifications were based on established risk criteria, including older age, lower education level (years) (< 12 years), financial hardship, living alone, history of falls, smoking, alcohol consumption, sleep disorders, physical inactivity, depressive symptoms, cognitive impairment, social isolation, and chronic diseases. Participants’ physical activity level^19^ and physical function^20^ were comparable to national averages for older adults in Japan.

**Table 1:** Participant characteristics.

Basic characteristics items	Cutoff point	Male (n = 111)	Cutoff point	Female (n = 131)
Age, years (mean, SD)		76.0 ± 5.0		74.0 ± 5.0
< 75, n (%)	< 75	46 (41.4)	< 75	63 (48.1)
≥ 75, n (%)	≥ 75	65 (58.6)	≥ 75	68 (51.9)
Height, cm (mean, SD)		164.0 ± 5.0		151.0 ± 5.0
Weight, kg (mean, SD)		65 ± 10.0		51 ± 10.0
BMI, kg/㎡ (mean, SD)		24 ± 3.0		22 ± 3.0
Individual factors				
Education level, years (mean, SD)		12.6 ± 2.4		11.8 ± 2.2
≥ 12, n (%)	≥ 12	92 (83.6)	≥ 12	100 (76.3)
< 12, n (%)	< 12	18 (16.4)	< 12	31 (23.7)
Economic status (poor), n (%)		16 (14.7)		8 (6.2)
Living alone, n (%)		4 (3.7)		21 (16.2)
PASE total score, median (IQR)		125.4 (94.8–154.0)		120.0 (94.7–155.4)
≥ 121.0, n (%)	≥ 121.0	49 (50)	≥ 122.9	58 (50)
< 121.0, n (%)	< 121.0	49 (50)	< 122.9	58 (50)
Leisure-time PA score, median (IQR)		21.2 (8.6–38.1)		15.0 (8.6–29.9)
≥ 17.6, n (%)	≥ 17.6	50 (49.9)	≥ 17.3	60 (49.6)
< 17.6, n (%)	< 17.6	51 (50.1)	< 17.3	61 (50.4)
Household PA score, median (IQR)		83.0 (60.3–106.0)		105.0 (70.0–106.0)
≥ 91, n (%)	≥ 91	54 (50)	≥ 105.0	58 (47.1)
< 91, n (%)	< 91	54 (50)	< 105.0	65 (52.9)
Work-related PA score, median (IQR)		0 (0–12.0)		0 (0–3.0)
≥ 1.5, n (%)	≥ 1.5	43 (38.7)	≥ 3.0	35 (27.1)
< 1.5, n (%)	< 1.5	68 (61.3)	< 3.0	94 (72.9)
Five cog score (mean, SD)		77.5 ± 18.0		83.9 ± 17.9
≥ 84, n (%)	≥ 84	54 (49.5)	≥ 83	64 (50.0)
< 84, n (%)	< 84	55 (50.5)	< 83	64 (50.0)
LSNS-6, score (mean, SD)		17.1 ± 6.0		19.3 ± 5.0
No social isolation, n (%)	≥ 12	92 (82.9)	≥ 12	116 (89.2)
Social isolation, n (%)	< 12	19 (17.1)	< 12	14 (10.8)
Performance test items				
Grip strength, kg (mean, SD)		33.0 ± 5.9		22.1 ± 3.3
Sit-and-reach test, cm (mean, SD)		35.5 ± 9.4		41.4 ± 9.8
TUG, second (mean, SD)		5.6 ± 1.0		5.8 ± 1.1
5-m habitual walk, second (mean, SD)		3.5 ± 0.5		3.4 ± 0.5
Hand working with pegboard, second (mean, SD)		37.2 ± 4.7		34.5 ± 3.6

SD, standard deviation; BMI, body mass index; PASE, Physical Activity Scale for Elderly; GDS, geriatric depression scale; LSNS, Lubben social network scale; TUG, timed up and go test; Five cog, five cognitive function; IQR, interquartile range.

Cutoff point: *Age*: Participants were divided into two groups: 65–74 years (< 75) and ≥ 75 years (≥ 75). *Education level*: Participants were categorized into two groups: less than 12 years (< 12) and 12 years or more (≥ 12) of education. *PASE total score*: Participants were divided into two groups based on the median value (male: 121.0, female: 122.9). *Leisure-time PA score*: Participants were categorized as below or above the median (male: 17.6, female: 17.3). *Household PA score*: Participants were categorized using the median score (male: 91.0, female: 105.0). *Work-related PA score*: Participants were classified as < 1.5 or ≥ 1.5 (male), and < 3.0 or ≥ 3.0 (female). *Five cognitive function score (Five cog)*: Participants were divided into two groups based on the average score (male: 84, female: 83). *LSNS-6*: A score below 12 indicates social isolation.

Tables 2, 3, and 4 present unstandardized regression coefficients, 95% confidence intervals (CIs), and p-values calculated using linear mixed-effects models that included interaction terms between each dichotomized factor and year. In these models, the year 2019 and the low-risk group were used as reference categories.

**Table 2:** Age, education level, living alone, and interaction (group × years).

Male (N = 111)		Age				Education Level				Living Alone*			
Performance Test Items	Year	Coefficient (*B*)	95% Cl		*P*	Coefficient (*B*)	95% Cl		*P*	Coefficient (*B*)	95% Cl		*P*
		Factor × Year	Lower	Upper		Factor × Year	Lower	Upper		Factor × Year	Lower	Upper	
Grip strength, kg	2020	0.16	− 1.22	1.54	0.822	− 0.09	− 1.88	1.70	0.921				
	2021	− 0.75	− 2.39	0.89	0.369	− 0.13	− 2.38	2.12	0.912				
Sit-and-reach test, cm	2020	− 1.54	− 4.48	1.40	0.305	− 0.20	− 3.75	3.36	0.914				
	2021	− 2.07	− 6.40	2.26	0.349	− 2.12	− 6.60	2.36	0.354				
TUG, seconds	2020	0.13	− 0.18	0.45	0.395	0.17	− 0.30	0.65	0.477				
	2021	0.18	− 0.14	0.49	0.271	**0.62**	**0.22**	**1.02**	**0.003**				
5-m habitual walk, seconds	2020	− 0.06	− 0.26	0.13	0.511	− 0.14	− 0.40	0.12	0.303				
	2021	0.19	− 0.06	0.44	0.140	− 0.15	− 0.44	0.14	0.307				
Hand working with pegboard, seconds	2020	2.17	0.41	3.92	0.016	− 1.35	− 3.04	0.34	0.119				
	2021	2.64	− 0.31	5.59	0.079	− 1.68	− 4.14	0.78	0.181				

Female (N = 131)		Age				Education Level				Living Alone			
Performance Test Items	Year	Coefficient (*B*)	95% Cl		*P*	Coefficient (*B*)	95% Cl		*P*	Coefficient (*B*)	95% Cl		*P*
		Factor × Year	Lower	Upper		Factor × Year	Lower	Upper		Factor × Year	Lower	Upper	
Grip strength, kg	2020	− 0.44	− 1.41	0.53	0.373	− 0.26	− 1.33	0.80	0.627	0.10	− 1.73	1.92	0.918
	2021	− 0.19	− 0.99	0.60	0.631	0.50	− 0.88	1.87	0.479	0.21	− 0.55	0.97	0.584
Sit-and-reach test, cm	2020	0.36	− 2.11	2.84	0.773	− 0.02	− 2.57	2.53	0.990	1.51	− 1.39	4.42	0.308
	2021	0.76	− 2.83	4.35	0.678	0.17	− 4.26	4.61	0.939	1.57	− 2.51	5.65	0.452
TUG, seconds	2020	0.09	− 0.12	0.30	0.414	0.28	0.00	0.55	0.049	0.02	− 0.38	0.42	0.935
	2021	0.06	− 0.18	0.29	0.647	0.19	− 0.12	0.50	0.218	− 0.16	− 0.42	0.11	0.254
5-m habitual walk, seconds	2020	0.10	− 0.07	0.27	0.235	0.20	0.00	0.41	0.050	− 0.26	− 0.46	− 0.07	0.008
	2021	− 0.03	− 0.22	0.15	0.734	0.09	− 0.12	0.30	0.408	**− 0.38**	**− 0.60**	**− 0.17**	**0.000**
Hand working with pegboard, seconds	2020	0.55	− 0.50	1.59	0.306	1.28	− 0.20	2.76	0.091	0.81	− 0.63	2.26	0.269
	2021	0.18	− 0.94	1.31	0.747	0.26	− 0.97	1.48	0.681	− 0.42	− 1.93	1.09	0.586

B: Unstandardized coefficients; CI: Confidence interval; TUG: Timed Up and Go test; LSNS: Lubben Social Network Scale; PASE: Physical Activity Scale for the Elderly; Five cog: Five cognitive function score.

All models included the year 2019 and the low-risk group as reference categories.

In male participants, “living alone” was treated as an adjustment variable due to the small number of individuals living alone (n = 4).

**Table 3:** Cognitive function and social interaction: Interaction (group × year).

Male (N = 111)		Five Cog Scores				LSNS			
Performance Test Items	Year	Coefficient (*B*)	95% Cl		*P*	Coefficient (*B*)	95% Cl		*P*
		Factor × Year	Lower	Upper		Factor × Year	Lower	Upper	
Grip strength, kg	2020	− 0.01	− 1.38	1.36	0.989	0.40	− 1.80	2.60	0.721
	2021	− 0.69	− 2.40	1.02	0.428	1.13	− 0.86	3.11	0.266
Sit-and-reach test, cm	2020	2.03	− 0.83	4.89	0.165	− 3.24	− 7.32	0.84	0.120
	2021	0.58	− 3.74	4.89	0.794	− 1.45	− 7.72	4.82	0.651
TUG, seconds	2020	− 0.02	− 0.32	0.28	0.890	0.34	− 0.20	0.88	0.212
	2021	0.08	− 0.24	0.39	0.620	0.10	− 0.29	0.50	0.600
5-m habitual walk, seconds	2020	0.05	− 0.14	0.25	0.586	0.18	− 0.06	0.43	0.140
	2021	0.25	− 0.01	0.52	0.060	− 0.30	− 0.54	− 0.06	0.016
Hand working with pegboard, seconds	2020	0.83	− 1.09	2.75	0.397	− 0.31	− 2.80	2.18	0.805
	2021	− 0.11	− 3.27	3.05	0.945	3.32	− 5.16	11.80	0.443

Female (N = 131)		Five Cog Scores				LSNS			
Performance Test Items	Year	Coefficient (*B*)	95% Cl		*P*	Coefficient (*B*)	95% Cl		*P*
		Factor × Year	Lower	Upper		Factor × Year	Lower	Upper	
Grip strength, kg	2020	− 0.19	− 1.16	0.77	0.695	− 0.64	− 1.47	0.18	0.124
	2021	0.29	− 0.52	1.09	0.490	− 0.34	− 1.47	0.79	0.553
Sit-and-reach test, cm	2020	0.10	− 2.38	2.59	0.934	− 2.04	− 6.32	2.25	0.351
	2021	− 1.49	− 5.29	2.31	0.443	− 6.33	− 14.56	1.89	0.131
TUG, seconds	2020	0.02	− 0.20	0.23	0.884	− 0.14	− 0.47	0.18	0.395
	2021	0.07	− 0.18	0.32	0.572	− 0.26	− 0.86	0.33	0.388
5-m habitual walk, seconds	2020	0.04	− 0.13	0.20	0.684	− 0.01	− 0.50	0.47	0.954
	2021	0.07	− 0.13	0.27	0.479	0.05	− 0.29	0.40	0.757
Hand working with pegboard, seconds	2020	0.44	− 0.62	1.51	0.416	1.28	0.29	2.27	0.011
	2021	− 0.07	− 1.20	1.06	0.904	1.24	− 1.05	3.54	0.289

B, unstandardized coefficients; CI, confidence interval; TUG, timed up and go test; LSNS, Lubben Social Network Scale; PASE, Physical Activity Scale for the Elderly; Five cog, five cognitive function score.

All models included the year 2019 and the low-risk group as reference categories.

**Table 4:** Physical activities: Interaction (group × year).

Male (N = 111)		PASE Total Score				Leisure-Time PA Score				Household PA Score				Work-Related PA Score			
Performance Test Items	Year	Coefficient (*B*)	95% Cl		*P*	Coefficient (*B*)	95% Cl		*P*	Coefficient (*B*)	95% Cl		*P*	Coefficient (*B*)	95% Cl		*P*
		Factor × Year	Lower	Upper		Factor × Year	Lower	Upper		Factor × Year	Lower	Upper		Factor × Year	Lower	Upper	
Grip strength, kg	**2020**	− 0.30	− 1.73	1.12	0.676	0.55	− 0.84	1.94	0.437	− 1.34	− 2.70	0.02	0.053	0.15	− 1.26	1.56	0.832
	**2021**	0.65	− 1.10	2.39	0.469	− 0.69	− 2.37	0.99	0.422	− 0.01	− 1.66	1.65	0.995	0.23	− 1.56	2.02	0.802
Sit-and-reach test, cm	**2020**	− 1.32	− 4.36	1.72	0.394	− 0.13	− 3.19	2.93	0.933	− 0.43	− 3.32	2.46	0.770	− 0.37	− 3.36	2.63	0.810
	**2021**	− 0.60	− 5.04	3.83	0.789	1.88	− 2.50	6.27	0.400	0.30	− 3.92	4.51	0.890	1.10	− 3.22	5.42	0.617
TUG, seconds	**2020**	0.02	− 0.30	0.33	0.902	− 0.15	− 0.45	0.15	0.319	0.23	− 0.07	0.53	0.140	0.10	− 0.19	0.39	0.497
	**2021**	− 0.13	− 0.47	0.21	0.456	**− 0.49**	**− 0.82**	**− 0.16**	**0.004**	0.09	− 0.24	0.42	0.596	− 0.06	− 0.38	0.27	0.736
5-m habitual walk, seconds	**2020**	0.09	− 0.10	0.29	0.363	0.06	− 0.13	0.26	0.520	0.04	− 0.16	0.24	0.690	0.14	− 0.05	0.33	0.151
	**2021**	0.12	− 0.17	0.41	0.417	0.07	− 0.21	0.36	0.612	0.09	− 0.19	0.36	0.543	− 0.01	− 0.31	0.28	0.926
Hand working with pegboard, seconds	**2020**	0.14	− 1.97	2.26	0.896	1.06	− 0.98	3.10	0.307	0.24	− 1.71	2.19	0.810	0.50	− 1.29	2.30	0.582
	**2021**	1.66	− 1.98	5.31	0.372	1.04	− 2.36	4.44	0.548	1.94	− 1.57	5.45	0.278	0.89	− 2.10	3.87	0.560

Female (n = 131)		PASE Total Score				Leisure-Time PA Score				Household PA Score				Work-Related PA Score			
Performance test items	year	Coefficient (*B*)	95% Cl		*P*	Coefficient (*B*)	95% Cl		*P*	Coefficient (*B*)	95% Cl		*P*	Coefficient (*B*)	95% Cl		*P*
		Factor × Year	Lower	Upper		Factor × Year	Lower	Upper		Factor × Year	Lower	Upper		Factor × Year	Lower	Upper	
Grip strength, kg	2020	− 0.04	− 1.08	1.00	0.938	− 0.16	− 1.17	0.86	0.760	− 0.92	− 1.93	0.08	0.072	0.26	− 0.78	1.30	0.620
	**2021**	0.27	− 0.59	1.13	0.535	− 0.10	− 0.92	0.73	0.819	− 0.57	− 1.38	0.24	0.169	− 0.21	− 0.99	0.57	0.599
Sit-and-reach test, cm	**2020**	− 2.22	− 4.81	0.37	0.093	− 0.71	− 3.29	1.86	0.587	− 1.83	− 4.36	0.70	0.157	− 0.88	− 3.80	2.03	0.554
	**2021**	− 1.01	− 4.78	2.76	0.599	1.30	− 2.39	5.00	0.490	− 2.90	− 6.60	0.80	0.124	− 0.74	− 5.11	3.64	0.742
TUG, seconds	**2020**	0.07	− 0.15	0.29	0.526	− 0.05	− 0.26	0.16	0.663	0.04	− 0.19	0.26	0.753	− 0.03	− 0.27	0.22	0.836
	**2021**	0.29	0.06	0.52	0.015	0.01	− 0.23	0.25	0.929	0.00	− 0.24	0.24	0.997	− 0.05	− 0.33	0.23	0.709
5-m habitual walk, seconds	**2020**	0.00	− 0.17	0.17	0.990	0.11	− 0.05	0.28	0.170	− 0.03	− 0.22	0.15	0.719	− 0.01	− 0.22	0.20	0.906
	**2021**	− 0.01	− 0.20	0.18	0.916	− 0.02	− 0.20	0.17	0.872	− 0.13	− 0.32	0.06	0.174	− 0.15	− 0.35	0.06	0.163
Hand working with pegboard, seconds	**2020**	0.31	− 0.83	1.45	0.591	0.54	− 0.56	1.65	0.336	0.40	− 0.69	1.50	0.471	0.47	− 0.82	1.77	0.473
	**2021**	0.71	− 0.48	1.89	0.241	1.32	0.19	2.45	0.021	− 0.18	− 1.33	0.97	0.759	0.87	− 0.28	2.01	0.137

B, unstandardized coefficients; CI, confidence interval; TUG, timed up and go test; LSNS, Lubben Social Network Scale; PASE, Physical Activity Scale for the Elderly; Five cog, five cognitive function score.

All models included the year 2019 and the low-risk group as reference categories.

Among men, *a lower education level (years)*—defined as less than 12 years—was significantly associated with an increase in TUG time in 2021 (β = 0.62, 95% CI 0.22–1.02 s, *p* = 0.003). Additionally, men with *a lower leisure activity score* showed a significant decrease in TUG time in 2021 (β = – 0.48, 95% CI – 0.82 to – 0.16 s,* p* = 0.004), indicating relatively worse performance among those with *a higher leisure activity score* (β = 0.48, 95% CI 0.16–0.82 s, *p* = 0.004). These findings suggest that among men, both lower education level (years) and *higher pre-pandemic leisure activity score* were associated with a decline in functional mobility as measured by the TUG test. In addition, in men, the interaction between age and year showed a statistically significant association with performance in Hand working with pegboard in 2020 (β = 2.17, 95% CI 0.41–3.92, *p* = 0.016); however, this did not meet the Bonferroni-adjusted significance threshold (*p* < 0.0056) and should therefore be interpreted with caution.

Among women, *living alone* was associated with significantly improved 5-m walking times in both 2020 (β = –0.26, 95% CI: –0.46 to –0.07 s, *p* = 0.008) and 2021 (β = –0.38, 95% CI: – 0.60 to – 0.17 s,* p* < 0.001). Similarly, in women, the interaction between social isolation (LSNS) and year showed a trend toward significance in Hand working with pegboard performance in 2020 (β = 1.28, 95% CI 0.29–2.27, *p* = 0.011), but this also did not reach the Bonferroni-corrected significance threshold.

## Discussion

This study was a three-year longitudinal investigation aimed at identifying risk factors for physical function decline among community-dwelling older adults during the COVID-19 pandemic. To the best of our knowledge, this is the first longitudinal study to examine the association between individual characteristics and changes in physical function by gender throughout the pandemic period.

The main finding of this study is that the factors associated with changes in physical function differed by gender. Specifically, among men, a lower education level (years) (i.e., less than 12 years) and a higher pre-pandemic leisure activity score were significantly associated with a worsening in Timed Up and Go (TUG) performance. Among women, living alone was associated with improvements in 5-m habitual walking speed. This gender-specific pattern suggests that men and women may respond differently to behavioral restrictions, and that certain individual characteristics may either exacerbate or buffer against functional decline depending on the social and behavioral context.

In men, the association between lower education level (years) and physical decline has been reported in previous studies^21,22^, and prolonged TUG times have also been linked to advanced age, lower socioeconomic status, low physical activity, and overweight^23^. Furthermore, studies conducted during the pandemic have shown that men with lower education level and higher pre-pandemic physical activity levels experienced greater reductions in physical activity due to stay-at-home measures^24,25^. These findings indicate that men with lower education level were more likely to experience physical declines, particularly with the significant reduction in activity during the pandemic. The present finding that men with a higher leisure activity score showed a significant worsening in TUG performance suggests that a reduction in physical activity during the pandemic may have contributed to functional decline. On the other hand, the improvement in walking ability among women living alone may reflect a distinctive pattern of adaptation. While living alone has traditionally been considered a risk factor for poor health and functional decline among older adults^26^, recent longitudinal studies have reported that older women living alone maintain higher levels of physical activity compared to those living with family members^27^, and experience less decline in instrumental activities of daily living (IADL)^14^. This suggests that living alone may, in some cases, encourage the development of self-sustaining behaviors. It is possible that the necessity of managing daily life independently contributed to the maintenance of physical activity levels even under social restrictions. Furthermore, previous studies have pointed out that diminished physical function is more strongly associated with poor social networks than with living alone itself^28^. These findings suggest that older women living alone may have been able to maintain or even improve their physical function as long as they were not socially isolated.

Although the associations between age and Hand working with pegboard performance in men, and between social isolation and performance in women, did not reach the Bonferroni-corrected significance level, they may suggest potential trends related to age- or isolation-associated changes in fine physical function. These findings warrant further investigation in future longitudinal studies.

Taken together, the findings of this study suggest that individual factors such as education level (years), pre-pandemic physical activity habits—including leisure activity score—and living arrangements had diverse influences on changes in physical function during the COVID-19-related behavioral restrictions, and that these influences were gender-specific.

This study has several limitations. First, the physical function assessments primarily focused on components such as muscle strength, agility, balance, flexibility, and manual dexterity, and did not include important indicators such as cardiorespiratory endurance^29^. Second, the study population was limited to older adults living in rural areas, and caution is needed when generalizing the findings to individuals in urban areas who may have been more strongly affected by movement restrictions. Third, the participants were relatively healthy older adults who voluntarily underwent health examinations, and therefore, the external validity of the findings may be limited in populations with different functional levels.

Nevertheless, the results of this study provide useful insights for designing primary and tertiary prevention strategies to prevent physical function decline among older adults during public health emergencies such as the pandemic. In terms of primary prevention, efforts should be made to enhance health education targeting older adults with lower education level (years), who tend to be at higher risk of functional decline, by effectively communicating the importance of physical activity and concrete ways to remain active^30^. Given that walking ability improved among women living alone, it is suggested that maintaining an independent lifestyle may contribute to preserving physical function. Therefore, for older adults living alone, policies that respect autonomy while supporting moderate social connectedness—such as community centers or online interactions—may be effective for primary prevention^31^. In the medium to long term, creating supportive living environments that facilitate physical activity even under behavioral restrictions—such as exercise opportunities within the home or remote exercise instruction using ICT—will be increasingly important^32^.For tertiary prevention, targeted interventions are needed for individuals vulnerable to functional decline due to behavioral restrictions or lifestyle changes, such as men with lower education level (years) or those who had high leisure activity scores before the pandemic. Possible interventions include individual home visits by community comprehensive support centers, implementation of in-home exercise programs, and early detection and response to losses in activity habits^33,34^. Additionally, ICT-based support such as tele-rehabilitation and physical activity tracking apps may also be effective^35^.

These findings are expected to contribute to the development of personalized prevention strategies based on risk, and to promote the maintenance of older adults’ health during public health crises. Where appropriate, such strategies should also be considered for integration into administrative policy and community-based integrated care systems.

## Conclusion

This study identified gender-specific factors associated with changes in physical function among older adults during the COVID-19 pandemic. Among men, lower education level (years) and higher pre-pandemic physical activity levels were associated with greater functional decline, while among women, living alone was associated with improved walking ability. These findings highlight the importance of developing gender-sensitive strategies to support older adults during public health emergencies.

## Data Availability

The datasets used and/or analyzed in this study are available with the corresponding author upon reasonable request.
